# Prevalence and Atypical Clinical Characteristics of *NOTCH3* Mutations Among Patients Admitted for Acute Lacunar Infarctions

**DOI:** 10.3389/fnagi.2020.00130

**Published:** 2020-05-14

**Authors:** Takashi Okada, Kazuo Washida, Kenichi Irie, Satoshi Saito, Michio Noguchi, Tsutomu Tomita, Masatoshi Koga, Kazunori Toyoda, Shuhei Okazaki, Takashi Koizumi, Ikuko Mizuta, Toshiki Mizuno, Masafumi Ihara

**Affiliations:** ^1^Department of Neurology, National Cerebral and Cardiovascular Center, Osaka, Japan; ^2^Research Fellow of Japan Society for the Promotion of Science, Tokyo, Japan; ^3^NCVC Biobank, National Cerebral and Cardiovascular Center, Osaka, Japan; ^4^Department of Cerebrovascular Medicine, National Cerebral and Cardiovascular Center, Osaka, Japan; ^5^Department of Neurology, Osaka University Graduate School of Medicine, Osaka, Japan; ^6^Department of Neurology, Graduate School of Medical Science, Kyoto Prefectural University of Medicine, Kyoto, Japan

**Keywords:** CADASIL, CADASIL scale-J, *NOTCH3*, R75P mutation, lacunar infarction

## Abstract

**Objectives:** Cerebral autosomal dominant arteriopathy with subcortical infarcts and leukoencephalopathy (CADASIL) is the most common hereditary small vessel disease, with reported frequencies of 2-5/100,000 individuals. Recently, it has been reported that some patients with *NOTCH3* gene mutations show atypical clinical symptoms of CADASIL. Assuming that CADASIL is underdiagnosed in some cases of lacunar infarction, this study was designed to examine the prevalence of *NOTCH3* gene mutations in the patients at highest risk who were admitted for lacunar infarctions.

**Methods:** From January 2011 to April 2018, 1,094 patients with lacunar infarctions were admitted to our hospital, of whom 31 patients without hypertension but with white matter disease (Fazekas scale 2 or 3) were selected and genetically analyzed for *NOTCH3* gene mutations (Phase 1). Furthermore, 54 patients, who were 60 years or younger, were analyzed for *NOTCH3* mutations (Phase 2). *NOTCH3* exons 2–24, which encode the epidermal growth factor-like repeat domain of the *NOTCH3* receptor, were analyzed for mutations by direct sequencing of genomic DNA.

**Results:** Three patients presented *NOTCH3* p.R75P mutations: two in the Phase 1 and one in the Phase 2 cohort. Among patients aged 60 years or younger and those without hypertension but with moderate-to-severe white matter lesions, the carrier frequency of p.R75P was 3.5% (3/85), which was significantly higher than that in the Japanese general population (4.7KJPN) (odds ratio [95% CI] = 58.2 [11.6–292.5]). All three patients with *NOTCH3* mutations had family histories of stroke, and the average patient age was 51.3 years. All three patients also showed white matter lesions in the external capsule but not in the temporal pole. The CADASIL and CADASIL scale-J scores of the three patients were 6, 17, 7 (mean, 10.0) and 13, 20, 10 (mean, 14.3), respectively.

**Conclusion:** Among patients hospitalized for lacunar infarctions, the p.R75P prevalence may be higher than previously estimated. The *NOTCH3* p.R75P mutation may be underdiagnosed in patients with early-onset lacunar infarctions due to the atypical clinical and neuroimaging features of CADASIL. Early-onset, presence of family history of stroke, external capsule lesions, and absence of hypertension may help predict underlying *NOTCH3* mutations despite no temporal white matter lesions.

## Introduction

Cerebral autosomal dominant arteriopathy with subcortical infarcts and leukoencephalopathy (CADASIL) is an autosomal dominant disorder caused by mutations in the *NOTCH3* gene in chromosome 19p13 (Tournier-Lasserve et al., 1993; Joutel et al., 1996) and is the most common hereditary small vessel disease, with clinical frequencies of 2-5/100,000 individuals (Joutel et al., 1997; Rutten et al., 2016). Patients with CADASIL have various clinical symptoms, such as lacunar infarction, migraines, progressive cognitive decline, and psychiatric problems (Chabriat et al., 2009), and the most specific imaging feature of patients with CADASIL are bilateral white matter hyperintensities (WMHs), especially WMHs of anterior temporal pole on magnetic resonance imaging (MRI) (Chabriat et al., 1995, 1998; Coulthard et al., 2000; Auer et al., 2001; Tomimoto et al., 2006; Bersano et al., 2018). However, some patients with *NOTCH3* mutations do not show the typical clinical and imaging features of CADASIL (Kim et al., 2006; Mizuno et al., 2008; Sari et al., 2019). Furthermore, it has been recently clarified that the potential prevalence of CADASIL may be higher than previously estimated (Rutten et al., 2016). Assuming that *NOTCH3* gene mutations may be involved in some cases of lacunar infarction, a representative small vessel disease, this study was designed to investigate the prevalence and clinical characteristics of *NOTCH3* gene mutations in the patients at highest risk who were admitted for lacunar infarction, using a whole sequence analysis of *NOTCH3* genes.

## Methods

### Study Design and Participants

This single-center cross-sectional study was performed at the National Cerebral and Cardiovascular Center (NCVC) of Osaka, Japan, and conducted in accordance with Declaration of Helsinki standards and after approval by the local NCVC ethical committee (M29-117-3). All participants signed a comprehensive NCVC biobank consent form.

The subjects of this study were patients with lacunar infarction with hyperintense signal on MRI Diffusion-weighted imaging (DWI).

Lacunar infarction was divided into two types based on the diameter of the occluded vessels: lacunar infarction of 20 mm or less in diameter and penetrating artery occlusion stroke exceeding 20 mm in diameter. Lacunar infarction was defined as lesions of 20 mm or less in the brainstem or subcortex in the territory of a penetrating artery. In principle, there was no major artery stenosis and no embolic sources. Penetrating artery occlusion stroke was defined as lesions exceeding 20 mm in the brainstem or subcortex in the territory of a penetrating artery. In principle, there was no major artery stenosis and no embolic sources.

We defined the inclusion criteria for cohort 1 and 2 to evaluate the frequency of CADASIL in the patients with the highest risk because genetic testing of all 1,094 patients is impractical in terms of cost and time.

In this study, therefore, hypertension and aging were used as exclusion criteria for patient selection because these are the two strongest risk factors for lacunar infarction (Veglio et al., 2009; Regenhardt et al., 2018; Kalaria and Hase, 2019). In addition, white matter lesions were used as inclusion criteria for patient selection because white matter lesions were the most representative imaging finding of CADASIL (Chabriat et al., 1998; Koizumi et al., 2019).

Among patients with acute lacunar infarctions, patients without hypertension but with white matter disease (Fazekas scale 2 or 3) were selected and genetically analyzed for *NOTCH3* gene mutations, in order to strictly target patients with lacunar infarction of 20 mm or less in diameter (Phase 1). Furthermore, patients with acute lacunar infarctions or penetrating artery occlusion stroke aged 60 years old or younger were genetically analyzed for *NOTCH3* gene mutations in order to broadly target patients with lacunar infarctions or penetrating artery occlusion stroke (Phase 2).

**Figure 1:**
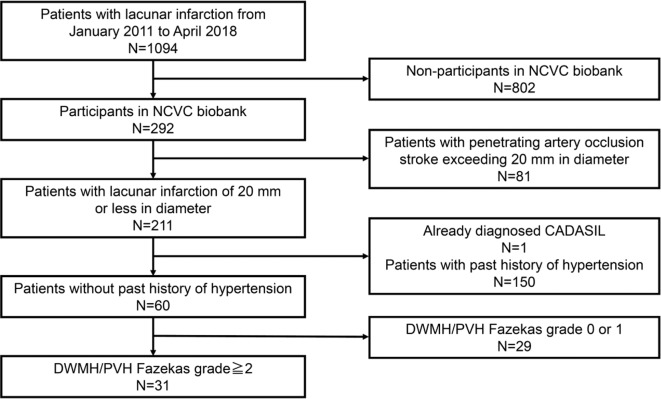
Patient selection flow diagram for the phase 1 cohort. NCVC, National Cerebral and Cardiovascular Center; CADASIL, Cerebral autosomal dominant arteriopathy with subcortical infarcts and leukoencephalopathy; DWMH, deep white matter hyperintensity; PVH, periventricular hyperintensity.

**Figure 2:**
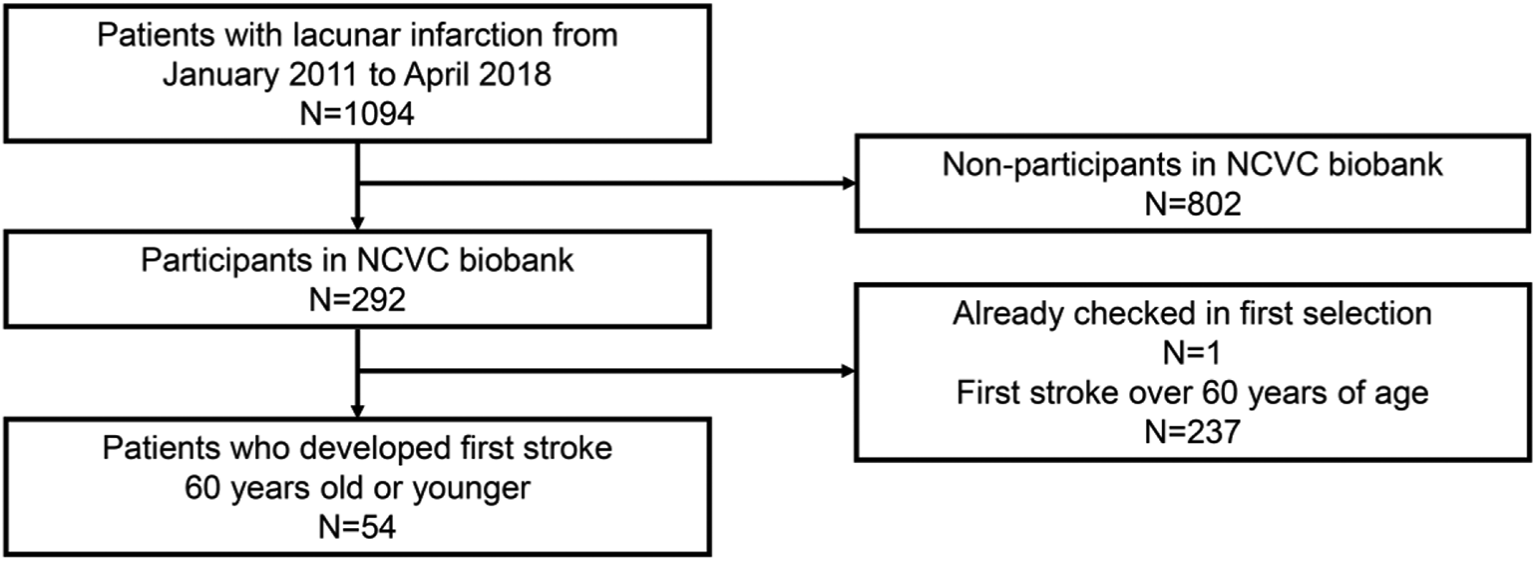
Patient selection flow diagram for the phase 2 cohort. NCVC, National cerebral and cardiovascular center.

Patients who satisfied the following criteria were included in this study:

- Phase 1 cohort: (1) patients who deposited samples in the NCVC biobank from January 2011 to April 2018, (2) patients with lacunar infarction (20 mm or less in diameter) without a past history of hypertension, and (3) patients with deep white matter hyperintensity (DWMH) or periventricular hyperintensity (PVH) of grade 2 or higher on the Fazekas scale (Fazekas et al., 1991).
- Phase 2 cohort: (1) patients who deposited samples in NCVC biobank from January 2011 to April 2018 and (2) patients with acute lacunar infarctions (20 mm or less in diameter) or penetrating artery occlusion stroke (exceeding 20 mm in diameter) who experienced their first stroke at the age of 60 years or younger.
- Lacunar infarction, penetrating artery occlusion stroke, and white matter lesions were diagnosed based on MRI. Patients who had already been diagnosed with CADASIL were excluded.
- A patient selection flow diagram is detailed in Figures 1, 2.

### Clinical Assessment

We collected clinical information that included data on clinical background (age at onset, sex, family history, and vascular risk factors), neurological symptoms, and MRI findings. The neurological symptoms that were evaluated included stroke, migraine, motor palsy, sensory disturbance, dizziness, pseudobulbar palsy, seizure, mood disturbance, and cognitive impairment.

Lacunar infarctions were diagnosed according to the Trial of Org 10172 Acute Stroke Treatment Criteria (Adams et al., 1993). Hypertension was defined as a blood pressure of at least 140/90 mmHg on two separate measurements or the use of antihypertensive drugs. Diabetes mellitus was defined as a fasting blood glucose level of at least 126 mg/dL, HbA1c 6.5% or higher, or the use of antidiabetic medications. Dyslipidemia was defined as a fasting serum low-density lipoprotein cholesterol level of at least 140 mg/dL or use of cholesterol-lowering therapy. Smoking status was determined by self-reporting as either a smoker (current or ex-smoker) or a nonsmoker. A family history of stroke was determined based on combined responses of the patient, sibling, and child to questions.

### Magnetic Resonance Imaging Protocol

Participants underwent imaging of the brain with a 3.0 Tesla MRI (Magnetom Verio or Spectra; Siemens Medical Solutions, Erlangen, Germany) scanner. A standardized clinical protocol was employed that included DWI and apparent diffusion coefficient (ADC), fluid-attenuated inversion recovery (FLAIR), T2 star weighted imaging (T2^*^WI), and MR angiography.

### Application of the CADASIL Scale and CADASIL Scale-J

The CADASIL scale of Pescini et al. (2012) is a simple scale that can be applied in a clinical setting as a screening tool for predicting a genetic diagnosis of CADASIL. The scale involves the additive score of 12 items (ranging from 0 to 25), whose cut-off value is 15. Result categories of the CADASIL scale were determined as positive (≥15) or negative (<15).

The CADASIL scale-J of Koizumi et al. (2019) is a modified, Japanese version of the CADASIL scale, with a score range from 0 to 25 and cut-off value of 16. This version uses eight items: hypertension, diabetes, young onset (≤ 50 years old), pseudobulbar palsy, stroke/TIA, family history, subcortical infarction, and temporal pole lesion.

### Genetic Examination

Genetic testing of *NOTCH*3 was performed as described previously (Mizuta et al., 2017). In brief, *NOTCH3* exons 2–24, which encode the epidermal growth factor-like repeat (EGFr) domain of the NOTCH3 receptor, were analyzed for mutations by direct sequencing of genomic DNA that was extracted from the peripheral blood. The sequence data were analyzed with SEQUENCHER (Gene Codes, HITACHI) to screen for mutations. Nucleotide substitutions were confirmed by restriction fragment length polymorphism analysis. We concluded that the variation was pathogenic when it was previously reported as pathogenic and/or when it resulted in a cysteine-related missense mutation in one EGFr.

## Results

### Baseline Demographics and Participants

During the study period, 1,094 patients with lacunar infarctions were admitted to the hospital (mean age ± standard deviation (*SD*), 72.0 ± 11.0; 61.0% men). In total, 292 of 1,094 patients participated in the NCVC biobank after providing written informed consent.

Of the overall cohort, 31 patients without hypertension but with white matter disease (Fazekas scale 2 or 3) were selected and genetically analyzed for *NOTCH3* gene mutations in the phase 1 cohort (mean age ± *SD*, 77.4 ± 9.6 11.0; 76.5% men) (Figure 1). The demographic data of the 31 patients in the phase 1 cohort are shown in Table 1.

**Table 1:** Demographic data of the 31 patients in the Phase 1 cohort.

**Demographics in 31 patients in Phase 1 cohort**	
Age, years (*SD*)	77.4 (9.6)
**Sex**	
Men, *n* (%)	23 (76.5%)
Women, *n* (%)	8 (23.5%)
Median NIHSS at baseline	3 (IQR 2–4)
HTN, *n* (%)	0 (0%)
DL, *n* (%)	17 (82.4%)
DM, *n* (%)	12 (40%)
Smoking, *n* (%)	14 (55.3%)

*SD, Standard deviation; NIHSS, national institute of health stroke scale; IQR, interquartile range; HTN, hypertension; DL, dyslipidemia; DM, diabetes mellitus*.

Furthermore, 54 patients, who were 60 years old or younger, were genetically analyzed for *NOTCH3* gene mutations in the phase 2 cohort (mean age ± *SD*, 53.0 ± 5.7; 77.8% men) (Figure 2). The demographic data of the 54 patients in the phase 2 cohort are shown in Table 2.

**Table 2:** Demographic data of the 54 patients in the Phase 2 cohort.

**Demographics in 54 patients in Phase 2 cohort**	
Age, years (*SD*)	53.0 (5.7)
**Sex**	
Men, *n* (%)	42 (77.8%)
Women, *n* (%)	12 (22.2%)
Median NIHSS at baseline	3 (IQR 2–4)
HTN, *n* (%)	45 (83.3%)
DL, *n* (%)	39 (72.2%)
DM, *n* (%)	22 (40.7%)
Smoking, *n* (%)	33 (61.1%)

*SD, Standard deviation; NIHSS, national institute of health stroke scale; IQR, interquartile range; HTN, hypertension; DL, dyslipidemia; DM, diabetes mellitus*.

### Prevalence of *NOTCH3* Mutations Among Patients With Lacunar Infarctions

Three patients had *NOTCH3* p.R75P mutations (ClinVar accession number: VCV000632306.1): two in the Phase 1 cohort (cases 1 and 2) and one in the phase 2 cohort (case 3). Among patients aged 60 years or younger or patients without hypertension with moderate-to-severe white matter lesions, the carrier frequency of p.R75P was 3.5% (3/85), which was significantly higher than that in the Japanese general population (4.7KJPN) (*n* = 4773) (odds ratio [95% CI] = 58.2 [11.6–292.5]) (Table 3). The p.R75P carrier frequency in biobank patients with lacunar infarction (1.0%, 3/292) was also significantly higher than that in 4.7KJPN (odds ratio [95% CI] = 16.5 [3.3–82.1]).

**Table 3:** Frequency of *NOTCH3* p.R75P mutations in patients and controls.

	**This study**	Japanese general population (4.7KJPN)^**^	**Japanese CADASIL patients**	
			**Ueda et al. (2015)**	**Koizumi et al. (2019)**
p.R75P carriers (*n*)	3	3	8	14
p.R75P non-carriers (*n*)	82	4770	62	139
Total (*n*)	85	4773	70	153
Frequency of p.R75P carrier (%)	3.5	0.06	11.4	9.2
	OR (95% CI)^*^			
	58.2 (11.6–292.5)			

* *Internet calculator (http://www.hutchon.net/ConfidOR.htm) was used*.

** *Number of p.R75P carriers among general Japanese population was estimated by its allele frequency (0.0003) and total number of participants (4773), because genotype frequency is not freely available on 4.7KJPN yet*.

*OR, Odds ratio; CI, confidence interval*.

The three patients with p.R75P mutations had no blood causality each.

### Clinical Characteristics of Patients With p.R75P Mutations

The clinical characteristics of patients with p.R75P mutations are shown in Table 4. The average patient age was 51.3 years. All patients had dyslipidemia and family histories of stroke; however, only the patient in case 2 showed cognitive impairment while the patient in case 3 had hypertension. No history of diabetes mellitus or cigarette smoking was found. Furthermore, the patients in case 1 and 3 had symptoms of dysarthria and dysesthesia but did not show typical clinical characteristics of CADASIL such as migraine and temporal pole lesions. The patient in case 2 showed relatively typical clinical symptoms, such as migraines and cognitive impairment.

**Table 4:** Clinical characteristics of patients with R75P mutations.

**Case**	**Age**	**Type of mutation**	**Stroke subtype**	**Symptoms**	**Migraine**	**Cognitive impairment**	**HTN**	**DL**	**DM**	**Smoking incidence**	**Family history of stroke**	**External capsule lesion**	**Temporal pole lesions**	**Cerebral microbleeds**	**Fazekas scale**	**CADASIL scale**	**CADASIL scale-J**
1	60s	R75P	SVD	Dysarthria, Dysesthesia	−	−	−	+	−	−	+	+	−	+	2	6	13
2	40s	R75P	SVD	Cognitive decline	+	+	−	+	−	−	+	+	−	+	3	17	20
3	50s	R75P	SVD	Vertigo, Dysarthria,	−	−	+	+	−	−	+	+	−	−	2	7	10

*Exact age is not shown for patient privacy*.

*HTN, Hypertension; DL, dyslipidemia; DM, diabetes mellitus; CADASIL, cerebral autosomal dominant arteriopathy with subcortical infarcts and leukoencephalopathy; SVD, small vessel disease*.

### Brain MRI Characteristics of Patients With p.R75P Mutations

**Figure 3:**
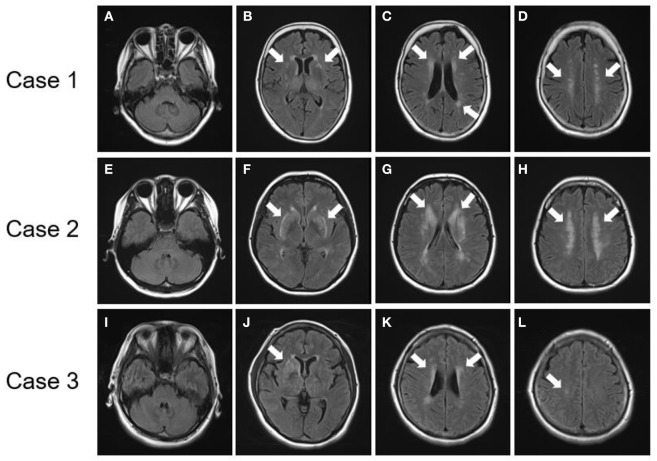
Brain MRI findings of patients with *NOTCH3* mutation. **(A–D)** FLAIR images of a patient with a *NOTCH3* p.R75P mutation (Case 1). **(E–H)** FLAIR images of a patient with a *NOTCH3* p.R75P mutation (Case 2). **(I–L)** FLAIR images of a patient with a *NOTCH3* p.R75P mutation (Case 3). **(A,E,I)** No patients presented lesions on their temporal pole lobes. **(B,F,J)** Arrows indicate hyperintensity lesions at external capsules. **(C,D,G,H,K,L)** Arrows indicate periventricular hyperintensity.

All three patients with p.R75P mutations had no temporal pole lesions (Figures 3A,E,I), while hyperintensity lesions were noticeable at the external capsule (Figures 3B,F,J), and the white matter lesions that were evaluated by the Fazekas scale were of grades 2-3 (Figures 3C,D,G,H,K,L). The patients in cases 1 and 2 exhibited several cerebral microbleeds, while the patient in case 3 had no cerebral microbleeds on T2^*^WI. MR angiography showed no arterial stenoses in all three patients with CADASIL.

### CADASIL Scale and CADASIL Scale-J Scores

The CADASIL and CADASIL scale-J scores of the three patients were 6, 17, 7 (mean, 10.0) and 13, 20, 10 (mean, 14.3), respectively (Table 4). The CADASIL scale-J score was higher than that of the CADASIL scale, although both scale scores were below their respective cut-off values (CADASIL scale: ≥15/25); CADASIL scale-J: ≥16/25).

## Discussion

In this study, the frequency of *NOTCH3* mutations was investigated for the first time in a Japanese lacunar stroke cohort; three patients had cysteine-sparing *NOTCH3* p.R75P mutations: two in the phase 1 cohort and one in the phase 2 cohort. The carrier frequency of p.R75P in this study was 3.5% (3/85), which was significantly higher than that in the Japanese general population (4.7KJPN) (odds ratio [95% CI] = 58.2 [11.6–292.5]). Furthermore, all three patients with *NOTCH3* mutations had family histories of stroke and showed hyperintensity lesions at external capsules and moderate-to-severe white matter lesions. However, these patients did not show typical CADASIL clinical and imaging features, such as temporal pole lesions. The CADASIL scale-J score was higher than that of the CADASIL scale.

CADASIL is the most common hereditary small vessel disease, and the estimated prevalence is 2-5/100,000 individuals in clinical practice (Joutel et al., 1997). The most frequent clinical symptoms are ischemic events (59%) and psychiatric disturbances (48%) (Bianchi et al., 2015). Migraine with aura and subcortical vascular cognitive impairment, which are associated with pseudobulbar palsy and urinary incontinence, are also characteristic symptoms of CADASIL (Chabriat et al., 2009). However, it has recently been reported that a subset of patients with *NOTCH3* gene mutations show atypical clinical symptoms of CADASIL, such as absence of temporal pole lesions (Ueda et al., 2015), elderly onset (Watanabe et al., 2012), and cerebellar atrophy (Sari et al., 2019). Furthermore, the exome aggregation consortium (ExAC) database study identified 206 EGFr cysteine-altering *NOTCH3* mutations in the ExAC, with an overall prevalence of 3.4/1,000 individuals (Rutten et al., 2016). The prevalence of CADASIL may be higher than previously thought; in fact, Dong Y et al. reported that one patient with a p.C697T mutation was found among 218 consecutive patients with lacunar infarction in the United Kingdom (Dong et al., 2003). Furthermore, Choi et al. reported that six patients with p.R544C mutations were found among 151 consecutive Korean patients with acute ischemic stroke (Choi et al., 2013), four of whom presented with large artery atherosclerosis and two with lacunar infarction. Our findings echo recent findings in Taiwan and the United Kingdom. Lee et al. reported that the *NOTCH3* p.R544C mutation was present in a significant number of individuals in Taiwan, including 60 of 7,038 healthy controls (0.9%), 17 of 800 patients with ischemic stroke (2.1%), and 16 of 245 patients with small vessel occlusion stroke (6.5%) from the Taiwan Biobank and that the other two cysteine-altering mutations (p.C853Y, and p.C884Y) were rarely detected (Lee et al., 2020). Furthermore, from the UK DNA Lacunar Stoke Study, Tan et al. identified single gene mutations in 14 patients (eight cysteine-altering *NOTCH3* variants in 11 patients, two *HTRA1* variants in two patients, and one missense *COL4A1* variant in one patient) among 950 patients with younger-onset apparently sporadic small vessel disease stroke using a targeted sequencing panel (14 of 950; 1.5%; Tan et al., 2019). These results strongly support our findings, which indicate that *NOTCH3* gene mutations may be involved in some cases of lacunar infarction, which is a representative small vessel disease. CADASIL is probably underdiagnosed in the wider stroke population.

In this study, all three patients with *NOTCH3* p.R75P mutations were relatively young and had family histories of stroke. Furthermore, all three patients showed hyperintensity lesions at external capsules and moderate-to-severe white matter lesions, but did not show temporal pole lesions. Temporal pole white matter lesions on MRI is one of the most characteristic imaging features of patients with CADASIL (Singhal et al., 2005; Yamamoto et al., 2009); however, some patients with CADASIL do not show temporal pole lesions. Abnormal temporal pole white matter lesions are less common in Asian patients than among Caucasians (Wang et al., 2011), and the prevalence of temporal pole involvement is only between 33.8–71% in the Japanese population (Ueda et al., 2015). In particular, cysteine-sparing *NOTCH3* missense mutations, such as p.R75P (Kim et al., 2006), p.R61W (Brass et al., 2009), p.D80G (Wollenweber et al., 2015), and p.R213K (Santa et al., 2003) are associated with atypical MRI findings with less anterior temporal lobe involvement (Matsushima et al., 2017; Muino et al., 2017). In a prior study of patients with CADASIL, 91% of cysteine-sparing *NOTCH3* mutations did not have anterior temporal pole involvement (Muino et al., 2017). Furthermore, the cysteine at codon 76 could be related to fixation of the double hairpin in the NOTCH3 EGFr, and the substitution of the basic arginine for a proline at codon 75 could induce a partial three-dimensional conformational change in the EGFr that leads to less involvement of the anterior temporal lobe (Mizuno et al., 2008). The precise mechanisms by which cysteine-sparing *NOTCH3* missense mutations induce CADASIL remains to be elucidated. We previously showed that temporal pole changes partially reflect dilated perivascular spaces (Yamamoto et al., 2009). Therefore, the p.R75P mutation may be less likely to dilate perivascular spaces than the cysteine-substitution mutations of *NOTCH3*. Cysteine-sparing mutations should be further studied to confirm their pathological role in CADASIL (Rutten et al., 2014; Coupland et al., 2018). Interestingly, CADASIL patients with *NOTCH3* p.R75P were frequently found throughout Japan. The allele frequency of p.R75P in the general population is 0.0003, according to the largest Japanese whole genome reference panel 4.7KJPN (https://jmorp.megabank.tohoku.ac.jp/202001/variants [accessed 25 March 2020], in the Japanese Multi Omics Reference Panel [jMorp], Tohoku Medical Megabank Organization).

It is of note that the carrier frequency of p.R75P in this study was 3.5% (3/85), which was significantly higher than that in 4.7KJPN (odds ratio [95% CI] = 58.2 [11.6–292.5]). The p.R75P carrier frequency in biobank patients with lacunar infarction (1.0%, 3/291) was also significantly higher than that in 4.7KJPN (odds ratio [95% CI] = 16.5 [3.3–82.1]). Our finding is compatible with the high frequency of p.R75P (9.2–11.4%) among Japanese CADASIL patients (Ueda et al., 2015; Koizumi et al., 2019). Mukai et al. analyzed the genotype–phenotype correlation based on the three most common mutations in Japanese CADASIL patients: the cysteine-sparing *NOTCH3* mutation p.R75P and cysteine-altering *NOTCH3* mutations p.R141C and p.R182C. p.R141C showed the most typical CADASIL phenotypes while p.R75P showed mild and atypical phenotypes, with a low frequency of temporal pole lesions, high frequency of hypertension, and low frequency of stroke/transient ischemic attack (TIA). Phenotypes of p.R182C were similar to those of p.R141C, except for a lower frequency of stroke/TIA. Initial symptoms of the 14 probands with p.R75P were none (four of 14; 28.6%), cognitive impairment (three of 14; 21.4%), depression (one of 14, 7.1%), and stroke/TIA (six of 14; 42.9%; Mukai et al., 2020). p.R75P presents as clinical symptoms that resemble sporadic lacunar infarcts, and head MRI often shows external capsule lesions and white matter lesions without temporal pole lesions (Kim et al., 2014; Ueda et al., 2015). Therefore, cysteine-sparing *NOTCH3* p.R75P mutation may be underdiagnosed in early-onset lacunar infarctions in daily clinical practice. Lacunar infarctions and white matter lesions of the external capsule, along with family history of stroke, can be prognostic factors that suggest the need for further genetic testing for possible underlying *NOTCH3* mutations.

*NOTCH3* genetic testing remains the diagnostic gold standard, and a clinical screening tool is eagerly desired because genetic testing is costly and time-consuming. Pescini et al. proposed the CADASIL scale, a screening tool that can be used to select patients for *NOTCH3* gene analysis (Pescini et al., 2012). The CADASIL scale showed high sensitivity in European studies; however, low sensitivity was reported in Chinese patients, possibly due to ethnic differences in clinical manifestations (Liu et al., 2015). Recently, Koizumi et al. developed the CADASIL scale-J, which can effectively discriminate between patients with CADASIL and patients with *NOTCH3*-negative CADASIL-like symptoms, among Japanese patients. The CADASIL scale-J exhibited a higher diagnostic accuracy than that of the CADASIL scale (Koizumi et al., 2019). The CADASIL scale-J was also well validated in 69 consecutive patients who underwent genetic testing. As a result, the sensitivity and specificity of the CADASIL scale-J were 78.9 and 85.7%, respectively (Koizumi et al., 2019). Furthermore, the CADASIL scale and the CADASIL scale-J scores of the patients with p.R75P were 6, 7, 17, and 10, 13, 20, respectively, in this study. While the CADASIL scale-J score was higher than that of the CADASIL scale, both scores were below their respective cut-off values (CADASIL scale: ≥15/25, CADASIL scale-J: ≥16/25). The low average scores in patients with p.R75P mutations may be due to a lower frequency of temporal pole lesions and older age onset compared with other CADASIL subtypes, such as cysteine-altering *NOTCH3* mutations (Ueda et al., 2015). The p.R75P mutation, which involves an arginine residue rather than a cysteine, is related to less frequent involvement of the anterior temporal area, thus hampering the clinical utility of CADASIL screening tools. It has been also reported that there is no single pathogenomic clinical or neuroimaging finding that can distinguish patients with CADASIL from those with sporadic stroke (Pantoni et al., 2010). Therefore, the present study supports the notion that clinicians should exercise caution concerning the possibility that atypical patients with *NOTCH3* mutations, such as p.R75P, may be unrecognized among patients with lacunar infarctions due to the atypical clinical and neuroimaging features of CADASIL.

From management and therapeutic perspectives, no definitive treatment has been established for CADASIL progression (Bersano et al., 2017). However, the early diagnosis and treatment for CADASIL, including the strict management of modifiable vascular risk factors, can improve patient prognosis. There are several reports about management options for CADASIL, and active smoking should be avoided, because it is independently associated with an earlier age of CADASIL onset and increased risk of stroke (Singhal et al., 2004; Chabriat et al., 2016). Additionally, the adequate control of homocysteine levels could decrease the risk of migraines (Singhal et al., 2004). Hypertension is also associated with an increased risk of stroke (Adib-Samii et al., 2010), cerebral microbleeds (Lee et al., 2017), intracerebral hemorrhage, and brain volume changes (Peters et al., 2006), as well as clinical progression. Other modifiable risk factors, such as diabetes mellitus (Viswanathan et al., 2006), alcohol abuse, and obesity, should also be controlled to prevent disease progression (Bersano et al., 2017). Furthermore, the administration of lomerizine, a diphenylmethylpiperazine Ca^2+^ channel blocker, could improve cognitive impairment and cerebral hypoperfusion in patients with CADASIL (Mizuno et al., 2009). Thus, the early detection of underdiagnosed CADASIL in patients who are initially admitted for lacunar infarctions should be carefully monitored to improve prognosis.

There are several limitations associated with the present study that warrant mentioning. First, this study is based on a relatively small number of patient because the genetic examination of all 1,094 patients was impractical in terms of cost and time. Patients aged 60–70 years and/or with white matter disease could be good targets for genetic testing (Tan et al., 2019; Lee et al., 2020). Age (60 years or younger), hypertension, and white matter lesions were used as criteria for patient selection in this study but these cohort selection criteria could be seen as arbitrary. Other characteristic findings of CADASIL, such as migraine, psychiatric symptoms, dementia, and family history of stroke could also be useful criteria for patient selection. More data are necessary to confirm the validity of our findings and their generalizability across other patient populations in the future. Second, this study was conducted at a single-center that was specialized for stroke and cardiovascular disease. Thus, selection biases may have led to an insufficient estimation of the frequency of *NOTCH3* gene mutations across more general populations. Further multi-center studies are needed to confirm the precise association of *NOTCH3* gene mutations with lacunar infarctions. Third, we only analyzed patients with lacunar infarctions in this study, and this could cause a bias in examining the exact prevalence of *NOTCH3* mutations among patients with overall subtypes of ischemic stroke, including large artery atherosclerosis, cardioembolism, and ischemic stroke of undetermined etiology. Further analyses including overall subtypes of ischemic stroke are necessary to confirm the exact prevalence of *NOTCH3* mutations in the future.

## Conclusion

Among patients hospitalized for lacunar infarction, the carrier frequency of p.R75P may be higher than previously estimated. Furthermore, the *NOTCH3* p.R75P mutation may be underdiagnosed in early-onset lacunar infarctions due to the atypical clinical and neuroimaging features of CADASIL. Early-onset, non-hypertensive lacunar infarcts and white matter lesions of the external capsule, and a family history of stroke are candidate factors that suggest the need for further genetic testing for underlying *NOTCH3* mutations.

## Data Availability Statement

The genetic data generated in this study can be obtained on NCBI ClinVar, https://www.ncbi.nlm.nih.gov/clinvar/, under accession no: VCV000632306.1.

## Ethics Statement

The studies involving human participants were reviewed and approved by Local ethics committee of NCVC. The patients/participants provided their written informed consent to participate in this study.

## Author Contributions

TO and KW: drafted the manuscript, designed the study, and acquired and analyzed the data. KI, SS, and MN: acquired and interpreted the data. SO, TT, MK, KT, and MI: revised the manuscript, interpreted the data, and supervised the study. TK, IM, and TM: performed the *NOTCH3* genetic examinations.

## Conflict of Interest

The authors declare that the research was conducted in the absence of any commercial or financial relationships that could be construed as a potential conflict of interest.
